# Senescence Is More Important in the Natural Lives of Long- Than Short-Lived Mammals

**DOI:** 10.1371/journal.pone.0012019

**Published:** 2010-08-06

**Authors:** Christopher Turbill, Thomas Ruf

**Affiliations:** Research Institute of Wildlife Ecology, University of Veterinary Medicine, Vienna, Austria; Texas A&M University, United States of America

## Abstract

**Background:**

Senescence has been widely detected among mammals, but its importance to fitness in wild populations remains controversial. According to evolutionary theories, senescence occurs at an age when selection is relatively weak, which in mammals can be predicted by adult survival rates. However, a recent analysis of senescence rates found more age-dependent mortalities in natural populations of longer lived mammal species. This has important implications to ageing research and for understanding the ecological relevance of senescence, yet so far these have not been widely appreciated. We re-address this question by comparing the mean and maximum life span of 125 mammal species. Specifically, we test the hypothesis that senescence occurs at a younger age relative to the mean natural life span in longer lived species.

**Methodology/Principal Findings:**

We show, using phylogenetically-informed generalised least squares models, a significant log-log relationship between mean life span, as calculated from estimates of adult survival for natural populations, and maximum recorded life span among mammals (R^2^ = 0.57, p<0.0001). This provides further support for a key prediction of evolutionary theories of ageing. The slope of this relationship (0.353±0.052 s.e.m.), however, indicated that mammals with higher survival rates have a mean life span representing a greater fraction of their potential maximum life span: the ratio of maximum to mean life span decreased significantly from >10 in short-lived to ∼1.5 in long-lived mammal species.

**Conclusions/Significance:**

We interpret the ratio of maximum to mean life span to be an index of the likelihood an individual will experience senescence, which largely determines maximum life span. Our results suggest that senescence occurs at an earlier age relative to the mean life span, and therefore is experienced by more individuals and remains under selection pressure, in long- compared to short-lived mammals. A minimum rate of somatic degradation may ultimately limit the natural life span of mammals. Our results also indicate that senescence and modulating factors like oxidative stress are increasingly important to the fitness of longer lived mammals (and vice versa).

## Introduction

Small rodents in captivity routinely reach ten times their mean life span in the wild. Why is it then that in human populations with an average life span of 40 to 80 years [1] nobody has ever lived to 400 years old or more? It seems that humans and perhaps other long-lived mammals grow old and die from intrinsic causes at a much younger age, relative to their mean natural life span, than shorter lived species. If this is true it implies that senescence has evolved to occur at an age of relatively high selection pressure in long-lived species [2]. It would also suggest that, even if senescence is detectable given a large enough sample-size [3], [4], [5], its relevance to fitness in natural populations may vary systematically among species according to their life span. Here, we address this question using a phylogenetically-informed comparative analysis of the relationship between maximum recorded life span and mean natural life span among mammal species.

The mean life span of adult individuals in a wild population, which can be calculated from estimates of survival probability, is a robust index of the rate of decline in selection pressure with age for species with determinate growth, like mammals. The strength of natural selection on genes expressed at different ages varies according to the probability of an individual surviving and its potential reproductive output at that age [6], [7], [8]. For mammals, selection pressure is greatest near the age of sexual maturity, when most individuals in a population contribute to the next generation, and declines thereafter in accordance with the rate of population attrition from environmental-caused mortalities.

Senescence is postulated to have evolved in the ‘selection shadow’ cast into old age. Genes with late-acting deleterious phenotypic effects may accumulate in the genome or such genes may be selected for because they also act to increase reproductive success at younger ages [7], [8], [9], [10]. As predicted by evolutionary theories of ageing, rates of senescence are correlated with variation in survival among populations [11], [12], [13]. Implicit in this theory, however, is that few individuals in natural populations survive to experience senescence; otherwise, senescence has evolved outside of a selection shadow. This condition is restated in seminal reviews, for example by Partridge and Barton [14] who speculated that, in pre-industrialised human populations “few individuals would have lived old enough to show evidence of ageing”, and Kirkwood and Austad [10] who stated: “As a rule, wild animals simply do not live old enough to grow old”. However, this traditional view is at odds with increasing evidence for senescence in natural populations of some species [3], [4], [5]. Comparative analyses of rates of senescence among birds and mammals have also questioned the assumption that a selection shadow is equally important to the evolution of senescence in all species. By modelling rates of senescence in survival using reconstructed life-history tables, Ricklefs [2], [12], [15] showed that the proportion of ageing-related mortalities is higher in bird and mammal species with lower initial mortality rates at maturity.

We re-address this question by comparing the mean and maximum life span of mammals – variables known for a much greater number of species. Mean natural life span, as explained above, is an index of the age-dependent reduction in selection pressure. Estimates of average adult survival rates are based chiefly on young individuals and therefore include few senescence-related mortalities. Any possible influence of senescence, however, would reduce estimates of survival for these species in our dataset. Maximum recorded life span represents the greatest recorded age of death for a species and reflects the timing of mortalities owing to intrinsic, biological causes (often in captive animals). Using an end-point method (maximum life span) to measure a rate (senescence) is obviously less accurate or informative than measuring the rate itself. Nevertheless, the large variation in maximum life span among mammal species is strongly correlated with rates of senescence in survival [16], justifying its widespread use in comparative studies.

Our analysis tests the hypothesis that the maximum life span, which provides an index of the age when senescence curtails longevity, is determined by the rate of reduction in selection pressure with age, which for mammals can be estimated by the mean life span of individuals in a population. If senescence has evolved under equally low selection pressure among all mammals, we expect a slope of zero in the relationship between the ratio of maximum/mean life span and mean life span; that is, maximum life span should represent a more or less constant function of mean life span. Variation in reproductive effort at various ages may also affect selection pressure in addition to survival rate [16]. We expect that, if anything, this should increase the slope of a relationship between maximum life span as a function of mean life span: short-lived species invest more in early reproduction and therefore may senesce at younger ages relative to their mean life span compared to long-lived species.

## Materials and Methods

We collated existing datasets to derive a median adult survival probability of natural populations [17], [18], [19] and maximum recorded life span [20] for 125 mammal species, including humans [1] (Table S1). We calculated mean life span (MLS) from survival probability (S) using the function: MLS = −1/ln(S).

To examine the relationship between mean and maximum life span, we fitted phylogenetically-informed generalized least squares (PGLS) models. Models were run using function gls in R version 2.9.2 [21]. In these models, we used an updated version [22] of the mammalian phylogenetic supertree [23] to set up correlation structures (Fig. S1) and implemented correlation classes available in the R-library ‘ape’ [24]. Initial trials (using both dated and equal branch length trees) showed that using the covariance matrix ‘corPagel’ [25], [26] resulted in the lowest estimates of model AIC (Akaike's information criterion). Maximum life span, mean life span, and their ratio were log transformed.

To further investigate the observed deviation (see Results) between observed and theoretically predicted maximum life span (assuming a constant ratio to mean life span) we analysed the difference between these (log transformed) measures as a function of their mean [27]. Plotting this relation, which is independent from regression estimates, indicated that differences between predicted and observed maximum life span were not normally distributed around zero but became increasingly negative as maximum life span increased, which was tested using a Runs test.

## Results

**Figure 1:**
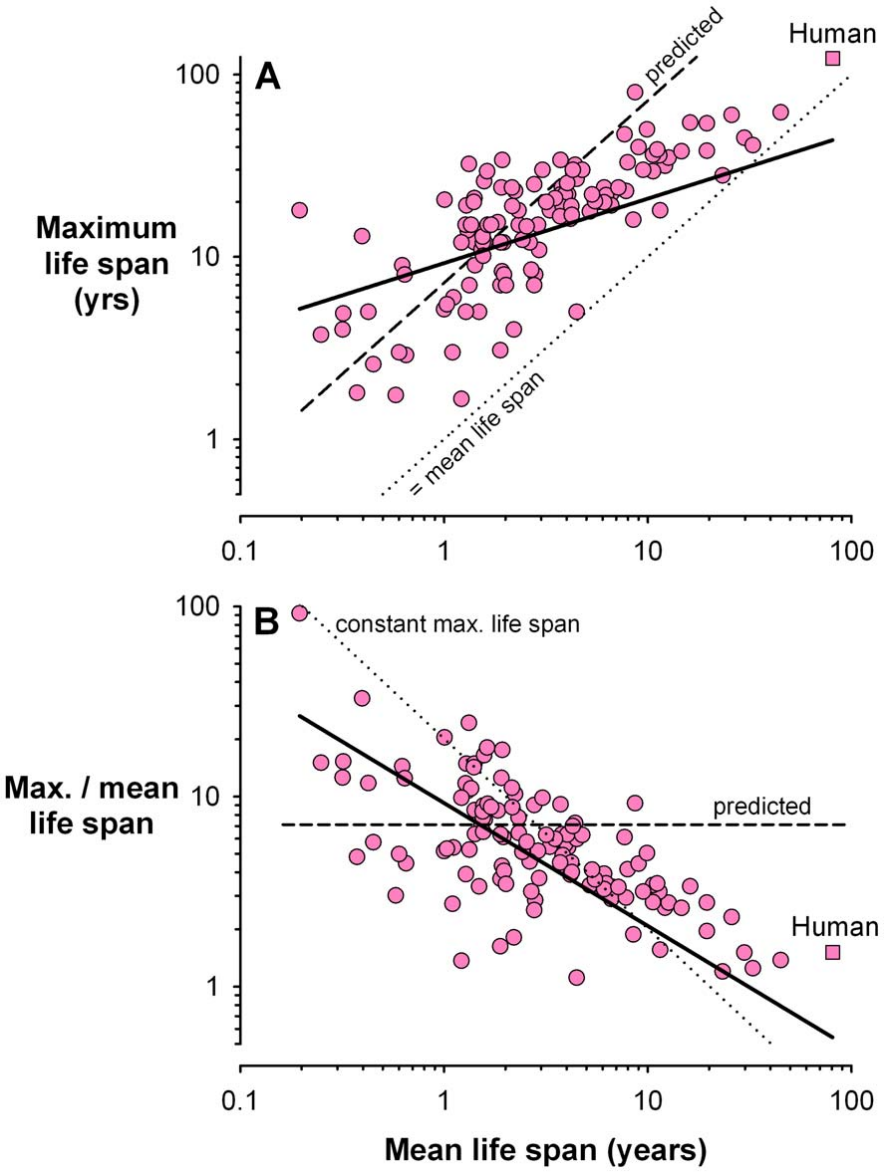
Mean and maximum life span of mammals. (**A**) Maximum recorded life span of 125 mammal species (*square*: humans) plotted as a function of their mean natural life span, calculated from estimates of annual survival. The observed slope of this relationship (*solid line*; 0.353±0.052) computed from phylogenetically informed generalized least squares (PGLS) differs significantly (t = 12.43, P<0.0001) from an average constant ratio between these two variables (*dashed line*). The maximum likelihood estimate of Pagel's λ in PGLS was 0.82 indicating a strong phylogenetic signal in maximum longevity among mammals. The deviation between predicted (*dashed line*) and observed maximum life spans (data points) was confirmed by a significant Runs test (Standardized Runs Statistic = −2.5248; P = 0.011) applied to the differences of these measures ordered along their means. (**B**) The same data plotted again to illustrate the pronounced decrease in the ratio of maximum to mean life span with increasing mean life span. The regression line (*solid line*; log10 ratio = 0.966−0.646× log10 mean life span) was computed using PGLS. The *dashed line* shows the prediction assuming an average constant ratio (maximum = 7.2× mean life span). A negative correlation is statistically expected when plotting a ratio as a function of its denominator using random, independent pairs of data [34]. For example, the *dotted line* shows the decrease in the ratio assuming a constant average maximum life span for all species (that is, if maximum life span was entirely independent of mean life span). This fact does not diminish the biological significance of relationships in empirical data [35], for which there is no mathematical or biological reason to exclude the alternative of no association (indeed this is the predicted relationship here).

We found a significant relation between mean and maximum life span among mammal species (R^2^ = 0.57, p<0.0001; Fig. 1A), supporting the hypothesis that rate of decline in selection pressure (as estimated by survival and mean life span) is a determinant of rate of senescence and hence maximum life span. In contrast to predictions of classical evolutionary theories of ageing, however, the slope of this relation (0.353±0.052 s.e.m.) indicated that maximum life span was not, on average, a constant multiple of mean life span. In fact, the ratio of maximum to mean life span decreased from >10 in short-lived to ∼1.5 in long-lived mammal species (Fig. 1B).

## Discussion

We show that mammals with higher survival probabilities have a mean life span in natural populations representing a greater fraction of their potential maximum life span. The ratio of mean to maximum life span can be interpreted as an index of the likelihood an individual will live to age when senescence begins to curtail their life span. Individuals of natural populations in which the mean life span is relatively near to the maximum ever recorded for the species clearly are more likely to experience senescence, that is, to die from intrinsic biological causes, than individuals of populations in which the mean life span occurs at a much younger age relative their potential maximum life span. A clear implication of our study, therefore, is that long-lived mammals are more likely than short-lived mammals to reach an age when their lives are affected by senescence (that is, an age closer to their maximum life span). In other words, our analysis suggests that senescence occurs at a much younger age, relative to the mean natural life span, in longer lived mammal species.

We believe a trivial explanation is highly unlikely for the relationship we observed between mean and maximum life span of mammals. Critically, there is no reason to suspect a bias towards underestimating the maximum life span of longer lived species. Estimates of maximum life span increase asymptotically with sample size. Maximum life span is estimated from comparably large sample sizes for the most long-lived species in our data set because these species (African elephant, horse, hippopotamus, gorilla, brown bear, donkey, baboon, capuchin monkey, and zebra) are routinely kept in captivity, domesticated or well-studied in the wild. For example, there are approximately 370 African elephants, 382 hippopotami and 512 brown bears currently living at institutes registered with the International Species Information System (ISIS), which suggests a sample size of ≫1000 for these species (as maximum ages were recorded over decades). Also, the influence of sample size (N) on maximum life span has been shown to be small (∼ln(ln(N)) and to decrease rapidly with increasing sample size [28]. It is highly unlikely, therefore, that the true maximum life span of long-lived mammals is near the extreme longevity suggested by the ratio with mean life span found among short-lived mammal species. It is also possible to show that the relationship we report is not an artefact caused by differences in the demographic shape of populations between short- and long-lived mammals.

Our results provide independent support for Ricklefs' [2], [12] conclusion that long-lived species suffer more age-related mortalities. Previously, Botkin and Miller [29] had pointed out the discrepancy between predicted maximum life span based on adult survival rates and actual recorded maximum life span in long-lived birds, which they argued was strong evidence for age-dependent mortality in wild populations of these species. Age at the onset of senescence and maximum life span have also been related to indices of reproductive effort, which themselves are correlated with survival, among bird and mammal species. These relationships have slopes of less than one, suggesting that, in accordance with the results of our analysis, species with longer generation times and slower rates of reproduction, which generally also live longer, suffer from senescence in survival at relatively younger ages [5], [30]. Thus, several lines of evidence, including our phylogenetically informed comparison of mean versus maximum life span, support an increasing role of senescence in the natural lives of longer living mammals.

**Figure 2:**
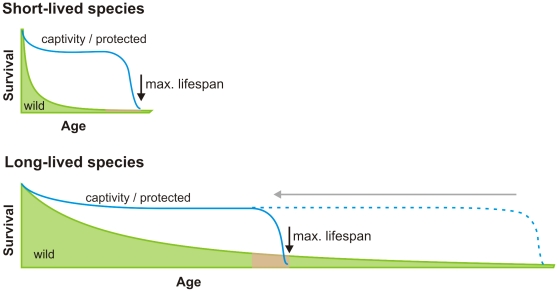
Schematic of the age of senescence and maximum life span in short- versus long-lived species. Schematic representation of survival curves for populations in the wild and in captivity or protected environments. For short-lived mammal species, senescence (shown as red shading) and therefore maximum life span occur at an age when most individuals in wild populations have succumbed to environmental mortalities; whereas, for long-lived species, senescence occurs at an earlier age relative to survival in the wild and hence at an age when selection pressure could remain high.

An age when senescence retards survival (i.e. near to the maximum life span) is reached by a higher proportion of individuals, and therefore remains under increasingly high selection pressure, in natural populations of longer lived mammal species (Fig. 2). This implies a minimum rate of senescence has been unavoidable in the evolution of mammals and could place a limit on their maximum life span, preventing humans from reaching Methuselah-like ages. Because senescence affects survival in long-lived species despite relatively strong opposing selection pressure, they have probably evolved mechanisms to delay its negative effects. Retarding senescence further seems to be unavailable to natural selection. More likely, such mechanisms probably involve negative trade-offs with other life history traits (e.g. reproductive effort) that make them untenable for natural populations [2]. Thus, any treatment that extends the life span of short-lived model organisms, like mice, may act on anti-senescence mechanisms that already operate at maximum capacity in long-lived species like humans.

Our findings make the clear prediction that senescence and modulating factors such as oxidative stress are more (or only) important to the fitness of long-lived mammals. Individuals of short-lived species are most likely to die at an age representing a small fraction of their potential maximum life span, suggesting their survival is largely governed by environmental causes of mortality. Most mammals are small and short-lived. Even if senescence is documented in a very large sample of individuals in wild populations of these species, it is probably of little consequence to fitness. In accordance, short-lived mammals exhibit ‘fast’ life-histories associated with relatively high rates of oxidative stress and somatic damage. The natural life span of long-lived mammal species, in contrast, is more likely to be curtailed by senescence [this study; 2]. Consequently, indirect factors like oxidative damage that may hasten biological ageing are more important in wild populations of these species. A decreasing ratio of maximum to mean life span in longer lived mammals clearly suggests a positive relationship between the fitness effects of senescence and life span among mammals.

**Figure 3:**
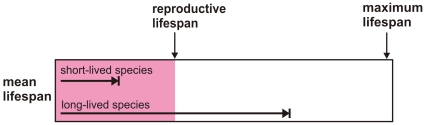
Schematic of mean life span in short- versus long-lived species. Schematic representation of the mean life span of wild populations as a function of potential reproductive and maximum life span (as exhibited in captive or protected populations) for short- and long-lived species.

Finally, because long-lived mammals naturally live a greater proportion of their potential maximum life span, they are more likely to exhibit senescent phenotypical traits (Fig. 3). For example, Jones et al. [3] could detect reproductive senescence in large but not small (and mostly short-lived) mammal species in their dataset. Most mammals exhibit a post-reproductive life span when kept in captivity, where all species live a large proportion of their maximum life span. Female laboratory mice and humans both accomplish around 75% of their reproductive output at an age representing 30% of their maximum life span [31]. Yet, unlike humans, few mice in wild populations live to this age. This may provide a parsimonious non-adaptive explanation for a post-reproductive life span in wild populations of long- but not short-lived mammals, which assumes only that reproductive and somatic senescence are subject to independent selective pressures [32], [33].

## Supporting Information

Figure S1 Sub-tree showing the phylogenetic relationships of the species in our analysis.
(0.76 MB TIF)

Table S1 Survival, mean and maximumm lifespan of mammals.
(0.24 MB DOC)
