# Supplementary material for: Senescence Is More Important in the Natural Lives of Long- Than Short-Lived Mammals
Source: PLoS One. 2010 Aug 6;5(8):e12019. doi: 10.1371/journal.pone.0012019 (PMC2917356; doi:10.1371/journal.pone.0012019)
Supplement: Table S1 — Survival, mean and maximumm lifespan of mammals. (0.24 MB DOC) [file pone.0012019.s002.doc]

| **Order** | **Species** | **Mass g** | **Survival** | **Mean lifespan yr** | **Maximum lifespan yr** |
| --- | --- | --- | --- | --- | --- |
| Artiodactyla | Aepyceros melampus | 52300 | 0.83 | 5.20 | 17.8 |
| Artiodactyla | Alces alces | 481000 | 0.80 | 4.43 | 27.0 |
| Artiodactyla | Capra ibex | 69300 | 0.97 | 32.83 | 22.3 |
| Artiodactyla | Capreolus capreolus | 22400 | 0.78 | 4.00 | 17.0 |
| Artiodactyla | Cervus elaphus | 240000 | 0.80 | 4.48 | 26.8 |
| Artiodactyla | Cervus nippon | 53000 | 0.78 | 4.02 | 25.4 |
| Artiodactyla | Connochaetes taurinus | 196000 | 0.85 | 5.94 | 21.5 |
| Artiodactyla | Damaliscus lunatus | 131000 | 0.74 | 3.31 | 18.0 |
| Artiodactyla | Giraffa camelopardalis | 955000 | 0.91 | 10.60 | 36.3 |
| Artiodactyla | Hemitragus jemlahicus | 67900 | 0.85 | 6.24 | 21.8 |
| Artiodactyla | Hippopotamus amphibius | 1520000 | 0.94 | 16.16 | 54.5 |
| Artiodactyla | Hippotragus niger | 234000 | 0.86 | 6.63 | 22.3 |
| Artiodactyla | Kobus ellipsiprymnus | 202000 | 0.85 | 6.11 | 19.9 |
| Artiodactyla | Kobus kob | 79500 | 0.76 | 3.71 | 21.9 |
| Artiodactyla | Odocoileus hemionus | 83800 | 0.78 | 3.92 | 22.0 |
| Artiodactyla | Odocoileus virginianus | 75000 | 0.64 | 2.23 | 23.0 |
| Artiodactyla | Oreamnos americanus | 71300 | 0.86 | 6.63 | 19.2 |
| Artiodactyla | Ovis aries | 34200 | 0.46 | 1.29 | 19.2 |
| Artiodactyla | Ovis canadensis | 74600 | 0.85 | 6.11 | 24.0 |
| Artiodactyla | Ovis dalli | 69600 | 0.89 | 8.50 | 16.0 |
| Artiodactyla | Pecari tajacu | 21300 | 0.63 | 2.16 | 24.0 |
| Artiodactyla | Phacochoerus aethiopicus | 75600 | 0.77 | 3.83 | 18.8 |
| Artiodactyla | Procapra gutturosa | 28100 | 0.70 | 2.77 | 7.0 |
| Artiodactyla | Pseudois nayaur | 51800 | 0.77 | 3.79 | 24.0 |
| Artiodactyla | Rangifer tarandus | 108000 | 0.83 | 5.51 | 20.2 |
| Artiodactyla | Rupicapra rupicapra | 32800 | 0.78 | 4.07 | 22.0 |
| Artiodactyla | Sus scrofa | 84500 | 0.49 | 1.41 | 21.0 |
| Artiodactyla | Syncerus caffer | 593000 | 0.91 | 10.60 | 29.5 |
| Artiodactyla | Tayassu pecari | 31400 | 0.75 | 3.51 | 21.0 |
| Artiodactyla | Tragelaphus strepsiceros | 205000 | 0.88 | 7.82 | 23.0 |
| Carnivora | Acinonyx jubatus | 50500 | 0.79 | 4.22 | 19.0 |
| Carnivora | Ailuropoda melanoleuca | 118000 | 0.81 | 4.77 | 30.0 |
| Carnivora | Canis lupus | 35000 | 0.54 | 1.63 | 29.5 |
| Carnivora | Felis silvestris | 4490 | 0.60 | 1.93 | 34.0 |
| Carnivora | Helogale parvula | 285 | 0.71 | 2.93 | 10.9 |
| Carnivora | Lontra canadensis | 8090 | 0.70 | 2.77 | 25.0 |
| Carnivora | Lynx rufus | 6390 | 0.47 | 1.32 | 32.3 |
| Carnivora | Martes americana | 882 | 0.63 | 2.16 | 19.0 |
| Carnivora | Martes pennanti | 3750 | 0.53 | 1.56 | 10.1 |
| Carnivora | Martes zibellina | 1170 | 0.71 | 2.90 | 15.0 |
| Carnivora | Meles meles | 11900 | 0.79 | 4.17 | 16.2 |
| Carnivora | Mephitis mephitis | 2400 | 0.52 | 1.55 | 12.9 |
| Carnivora | Mungos mungo | 1260 | 0.68 | 2.63 | 12.0 |
| Carnivora | Mustela nigripes | 907 | 0.60 | 1.96 | 12.0 |
| Carnivora | Mustela putorius | 984 | 0.53 | 1.56 | 14.0 |
| Carnivora | Nyctereutes procyonoides | 4220 | 0.47 | 1.31 | 14.0 |
| Carnivora | Panthera leo | 159000 | 0.90 | 9.49 | 30.0 |
| Carnivora | Procyon lotor | 6370 | 0.37 | 1.01 | 20.6 |
| Carnivora | Taxidea taxus | 7840 | 0.53 | 1.58 | 26.0 |
| Carnivora | Urocyon cinereoargenteus | 3830 | 0.46 | 1.28 | 15.0 |
| Carnivora | Ursus americanus | 111000 | 0.80 | 4.41 | 32.0 |
| Carnivora | Ursus arctos | 196000 | 0.90 | 9.91 | 50.0 |
| Carnivora | Ursus maritimus | 375000 | 0.95 | 19.50 | 38.2 |
| Carnivora | Vulpes lagopus | 3600 | 0.56 | 1.70 | 15.0 |
| Carnivora | Vulpes vulpes | 4840 | 0.54 | 1.64 | 15.0 |
| Chiroptera | Carollia perspicillata | 19 | 0.66 | 2.42 | 12.4 |
| Chiroptera | Eptesicus fuscus | 17 | 0.49 | 1.40 | 20.0 |
| Chiroptera | Myotis leibii | 5 | 0.59 | 1.90 | 12.0 |
| Chiroptera | Myotis lucifugus | 8 | 0.77 | 3.73 | 34.0 |
| Chiroptera | Myotis myotis | 26 | 0.83 | 5.33 | 22.0 |
| Chiroptera | Myotis sodalis | 7 | 0.73 | 3.18 | 20.0 |
| Chiroptera | Pipistrellus pipistrellus | 5 | 0.76 | 3.71 | 16.7 |
| Chiroptera | Pipistrellus subflavus | 6 | 0.48 | 1.36 | 15.0 |
| Chiroptera | Plecotus auritus | 8 | 0.72 | 3.04 | 30.0 |
| Chiroptera | Rhinolophus ferrumequinum | 23 | 0.79 | 4.29 | 30.0 |
| Didelphimorphia | Didelphis virginiana | 2470 | 0.10 | 0.42 | 5.0 |
| Diprotodontia | Burramys parvus | 44 | 0.44 | 1.22 | 12.0 |
| Diprotodontia | Macropus robustus | 26000 | 0.87 | 7.18 | 24.0 |
| Diprotodontia | Onychogalea fraenata | 4940 | 0.80 | 4.48 | 5.0 |
| Diprotodontia | Trichosurus caninus | 3140 | 0.79 | 4.24 | 17.0 |
| Diprotodontia | Trichosurus vulpecula | 2700 | 0.68 | 2.54 | 14.7 |
| Insectivora | Talpa europaea | 85 | 0.47 | 1.33 | 7.0 |
| Lagomorpha | Lepus americanus | 1570 | 0.21 | 0.64 | 8.0 |
| Lagomorpha | Lepus europaeus | 3820 | 0.53 | 1.55 | 12.0 |
| Lagomorpha | Ochotona princeps | 158 | 0.61 | 2.02 | 7.0 |
| Lagomorpha | Oryctolagus cuniculus | 1590 | 0.01 | 0.20 | 18.0 |
| Lagomorpha | Sylvilagus floridanus | 1210 | 0.20 | 0.62 | 9.0 |
| Peramelemorphia | Isoodon macrourus | 1510 | 0.19 | 0.60 | 3.0 |
| Perissodactyla | Equus asinus | 180000 | 0.88 | 7.69 | 47.0 |
| Perissodactyla | Equus burchellii | 277000 | 0.90 | 9.01 | 40.0 |
| Perissodactyla | Equus caballus | 400000 | 0.98 | 44.95 | 62.0 |
| Perissodactyla | Equus hemionus | 236000 | 0.91 | 11.12 | 38.8 |
| Primates | Cebus olivaceus | 2800 | 0.97 | 32.83 | 41.0 |
| Primates | Chlorocebus aethiops | 4030 | 0.92 | 12.15 | 31.6 |
| Primates | Gorilla gorilla | 114000 | 0.95 | 19.50 | 54.0 |
| Primates | Macaca fascicularis | 4590 | 0.93 | 14.65 | 38.0 |
| Primates | Macaca fuscata | 10100 | 0.88 | 7.96 | 33.0 |
| Primates | Macaca mulatta | 6450 | 0.92 | 11.40 | 36.0 |
| Primates | Macaca sinica | 4660 | 0.92 | 12.65 | 35.0 |
| Primates | Microcebus murinus | 67 | 0.58 | 1.84 | 15.5 |
| Primates | Pan troglodytes | 45100 | 0.96 | 25.81 | 60.0 |
| Primates | Papio hamadryas | 16900 | 0.97 | 29.80 | 45.0 |
| Primates | Theropithecus gelada | 16000 | 0.96 | 23.31 | 28.0 |
| Proboscidea | Loxodonta africana | 3940000 | 0.89 | 8.66 | 80.0 |
| Rodentia | Apodemus flavicollis | 32 | 0.04 | 0.32 | 4.0 |
| Rodentia | Castor canadensis | 18100 | 0.65 | 2.33 | 15.0 |
| Rodentia | Cynomys ludovicianus | 797 | 0.69 | 2.68 | 8.5 |
| Rodentia | Dipodomys spectabilis | 125 | 0.40 | 1.10 | 3.0 |
| Rodentia | Eliomys quercinus | 115 | 0.38 | 1.03 | 5.5 |
| Rodentia | Erethizon dorsatum | 7420 | 0.65 | 2.32 | 18.0 |
| Rodentia | Glaucomys sabrinus | 138 | 0.08 | 0.40 | 13.0 |
| Rodentia | Glis glis | 128 | 0.49 | 1.42 | 9.0 |
| Rodentia | Hystrix africaeaustralis | 14900 | 0.84 | 5.81 | 20.0 |
| Rodentia | Liomys adspersus | 51 | 0.18 | 0.58 | 1.8 |
| Rodentia | Marmota flaviventris | 3710 | 0.70 | 2.80 | 8.0 |
| Rodentia | Marmota marmota | 4060 | 0.92 | 11.51 | 18.0 |
| Rodentia | Microtus oeconomus | 33 | 0.07 | 0.37 | 1.8 |
| Rodentia | Myocastor coypus | 6360 | 0.49 | 1.41 | 12.0 |
| Rodentia | Myodes gapperi | 20 | 0.44 | 1.22 | 1.7 |
| Rodentia | Myodes glareolus | 21 | 0.04 | 0.32 | 4.9 |
| Rodentia | Oryzomys megacephalus | 57 | 0.02 | 0.25 | 3.8 |
| Rodentia | Peromyscus maniculatus | 20 | 0.60 | 1.93 | 8.3 |
| Rodentia | Proechimys semispinosus | 359 | 0.11 | 0.45 | 2.6 |
| Rodentia | Rhabdomys pumilio | 41 | 0.22 | 0.65 | 2.9 |
| Rodentia | Sciurus carolinensis | 546 | 0.59 | 1.91 | 24.0 |
| Rodentia | Spermophilus armatus | 307 | 0.46 | 1.28 | 5.0 |
| Rodentia | Spermophilus beldingi | 272 | 0.52 | 1.53 | 11.0 |
| Rodentia | Spermophilus lateralis | 175 | 0.59 | 1.90 | 7.0 |
| Rodentia | Spermophilus richardsonii | 325 | 0.41 | 1.11 | 6.0 |
| Rodentia | Tachyoryctes splendens | 227 | 0.59 | 1.89 | 3.1 |
| Rodentia | Tamias amoenus | 51 | 0.37 | 1.00 | 5.2 |
| Rodentia | Tamias striatus | 112 | 0.60 | 1.97 | 8.0 |
| Rodentia | Tamiasciurus hudsonicus | 200 | 0.53 | 1.58 | 12.0 |
| Rodentia | Zapus hudsonius | 18 | 0.51 | 1.49 | 5.0 |
| Rodentia | Zapus princeps | 27 | 0.64 | 2.20 | 4.0 |
